# Lower extremity bilateral explosive strength asymmetry predicts non-contact low back injuries in mine rescue workers: a prospective cohort study

**DOI:** 10.3389/fpubh.2026.1740041

**Published:** 2026-02-02

**Authors:** Shuo Li, Sanjun Yang, Yunchen Meng

**Affiliations:** 1 Department of Physical Education and Research, China University of Mining and Technology-Beijing, Beijing, China; 2 Institute for Emergency Rescue Ergonomics and Protection, China University of Mining and Technology-Beijing, Beijing, China

**Keywords:** bilateral asymmetry, explosive strength, low back injury, mine rescue work, single-leg countermovement jump

## Abstract

**Objective:**

The purpose of this study was to investigate the relationship between bilateral lower extremity explosive strength asymmetry and non-contact low back injuries in mine rescue workers, a specialized occupational group.

**Methods:**

A single-leg countermovement jump (SLCMJ) test was performed on 101 rescue members who had participated in frontline rescue missions, using a force platform. The collected force platform data were used to calculate lower limb asymmetry. Injury incidence was measured by recording all non-contact low back injuries occurring during routine training or rescue operations over a 12-month follow-up period. Jump metrics (including eccentric peak force, vertical velocity at takeoff, peak takeoff acceleration, and takeoff peak force) associated with non-contact low back injuries were identified through Poisson regression analysis, and the optimal threshold for predicting injuries was determined using the receiver operating characteristic (ROC) curve.

**Results:**

Bilateral takeoff peak force asymmetry significantly increased the risk of non-contact low back injury. Each 1% increase in asymmetry raised the injury risk by 18.5% (RR = 1.185, 95% CI: 1.091–1.288, *p* < 0.001), equivalent to an absolute increase of 3.2% (RD = 0.032, 95% CI: 0.009–0.056). Using the optimal threshold of 7.05% for risk stratification, rescue workers in the high-risk group had a 3.6 times higher injury risk than the low-risk group (RR = 3.64, 95% CI: 1.353–9.832, *p* = 0.011), corresponding to an absolute risk difference of 24.1% (RD = 0.241, 95% CI: 0.073–0.409).

**Conclusion:**

The interlimb asymmetry measured during the single-leg countermovement jump can be used to predict the risk of non-contact low back injuries in mine rescue workers over a 12-month follow-up period. Particular emphasis should be placed on the interlimb asymmetry of takeoff peak force.

## 1 Introduction

In sports science, bilateral asymmetry in lower-limb function is recognized as a risk factor for sports injuries (1). This asymmetry is prevalent among athletes due to sport-specific movement patterns (e.g., kicking in football or shooting in basketball) and unilateral dominance training. Cheng et al. (2) indicated that functional limb asymmetry not only increases injury risk but can also be exacerbated by injuries themselves, creating a positive feedback loop that amplifies bilateral differences. Nie et al. (3) noted that muscular imbalances in one part of the human movement system inevitably lead to compensatory mechanisms in other areas to regulate the body’s center of gravity and maintain postural stability. Similarly, Petch et al. (4) suggested that both under- and over-activation of muscles can impair motor control. Thus, such muscle strength imbalances are both a manifestation of abnormal movement patterns and a factor that exacerbates them, leading to increased limb asymmetry. Numerous studies have investigated the use of bilateral lower-limb asymmetry to assess athletic performance. For example, Madruga-Parera et al. (5) identified a correlation between inter-limb differences and declines in performance among adolescent tennis players. Similarly, Bishop et al. (6) found that vertical and horizontal asymmetry in female soccer players was strongly negatively correlated with performance in jumping and sprinting. Furthermore, researches have reported the use of bilateral asymmetries across various functional tests as predictive risk factors for non-contact injuries: Read et al. (7) identified asymmetry in the peak vertical ground reaction force (pVGRF) during single-leg countermovement jumps (SLCMJ) as the most significant risk factor for lower-limb injuries in adolescent footballers. Smith et al. (8) reported that anterior (ANT) reach asymmetry in the Y-balance test was significantly associated with non-contact injuries, while Mokha et al. (9) found that asymmetric or low FMS scores were effective predictors of musculoskeletal injuries. However, findings across the literature are mixed, as the observed associations vary depending on the population, injury type, and asymmetry metric used. For example, Fort-Vanmeerhaeghe et al. (10) found that uninjured young team athletes exhibited lower asymmetry in SLCMJ height compared to their injured counterparts. In contrast, Brumitt et al. (11) found that jumping asymmetry was unrelated to injury.

In specialized professions such as emergency rescue, mine rescue personnel frequently operate in environments characterized by high physical exertion, elevated risks, and significant psychological pressure (12). During training and rescue operations, they perform demanding tasks such as load-bearing ascents, heavy lifting, and long-distance casualty transport. Their physical capabilities are critical determinants of both operational success and personal safety. However, Buoncristiani et al. (13) noted that over time, the combined effects of complex hazardous environments and unilateral movement patterns substantially increase the likelihood of asymmetry in this population. Supporting this, Miratsky et al. (14) observed in a study of firefighters that repetitive unilateral loading movements during training—such as carrying equipment and operating ladders—promote limb dominance and asymmetry. Furthermore, Vaulerin et al. (15) found, through Y-balance tests on 39 firefighters, that bilateral lower-limb asymmetry had high diagnostic value for injury prediction. Consequently, detailed research on limb asymmetry among mine rescue personnel is warranted. Wu et al. (16) observed that low back injuries accounted for the highest proportion (37.2%) of occupational injuries among mine rescue personnel, with most being non-contact injuries (e.g., strains and sprains). However, whether the occurrence of these prevalent injuries is associated with bilateral asymmetry in lower-limb explosive strength among rescue personnel remains to be investigated.

In study on identifying limb asymmetry in young female soccer players, Bishop et al. (6) suggested that vertical jumps might be more sensitive than horizontal ones for detecting asymmetry, and that unilateral jump tests are easy to administer and ecologically valid. Guan et al. (17) noted that unilateral jumping, which relies on power generated from one side, provides a more direct measurement of bilateral differences compared to bilateral jumping. Similarly, Benjanuvatra et al. (18) and Heil et al. (19) argued that unilateral support and propulsion are more prevalent in activities like sprinting, jumping, and directional changes—movements closely associated with rescue and training tasks for mine rescuers. Additionally, landing mechanisms in unilateral jumping are often linked to non-contact injury mechanisms such as anterior cruciate ligament (ACL) tears (20, 21).

Based on these findings, this study selected the SLCMJ test and employed a prospective cohort study design to determine the risk relationship between bilateral asymmetry in lower extremity explosive strength and non-contact low back injuries in mine rescuers over a 12-month follow-up period. The study hypothesized that greater interlimb asymmetry in takeoff peak force during the SLCMJ would be significantly associated with an increased risk of sustaining a non-contact low back injury in this occupational group.

## 2 Methods

### 2.1 Subjects

This study employed a prospective cohort design and utilized G*Power 3.1 software for statistical analysis. This statistical test is configured as log-binomial regression and Poisson regression within the z-test family. Based on the research (1, 22), G*Power 3.1 was used with the following parameters: test family = “z tests,” statistical test = “Poisson regression,” tail(s) = two, *α* error probability = 0.05, power (1 – *β* error probability) = 0.8, Exp(β) = 2, Base rate exp(*β*_0_) = 0.14. The primary outcome for which the effect size was calculated was the incidence of non-contact low back injury over 12 months. The calculated total sample size was 92 participants.

The study participants comprised 101 members of a Chinese national mine emergency rescue squadron who had participated in frontline rescue missions. After baseline testing, all participants entered a 12-month follow-up period. During this period, 14 participants (13.9%) were lost to follow-up due to reasons including technical issues (*n* = 8), failure to maintain contact (*n* = 6). Consequently, 87 participants (86.1%) completed the full one-year follow-up observation and were included in the final analysis. A post-hoc power analysis, based on the final sample of 87, the observed effect (RR = 1.185 per 1% increase in takeoff peak force asymmetry), and *α* = 0.05, confirmed the achieved power remained above 0.75, close to the preset target of 0.8. All participants provided voluntary informed consent after understanding the study’s purpose.

### 2.2 Procedures

The SLCMJ test was employed to evaluate bilateral asymmetry in lower limb explosive strength. Assessments were conducted on ForceDecks force plates (ForceDecks, Brisbane, Australia) at a sampling frequency of 1,000 Hz. The force plates provided raw vGRF data, from which the system’s proprietary software automatically calculated relevant kinematic data, including eccentric peak force (N), concentric peak force (N), peak vertical velocity at takeoff (m/s), peak takeoff acceleration (m/s^2^), and jump height (m), etc. Prior to testing, participants performed a standardized 10-min dynamic warm-up, consisting of jogging, leg swings (forward/sideward), bodyweight squats, lunges.

At the start of the test, participants stood on the center of the force plate in a single-leg stance, with hands on their hips (palms facing inward) and eyes looking forward. Upon hearing the “Start” command, they rapidly performed a deep squat to a self-selected depth, followed by a swift lower limb extension to execute a maximal vertical jump. Throughout the movement, participants maintained body stability. After landing, they remained standing on the force plate for 1–2 s.

Before formal testing, each participant performed two practice trials, followed by three formal assessment trials. The average score from the three formal trials was recorded, with a one-minute rest period between each trial. All data collection was performed by the same researcher. Participants received pre-test training in the SLCMJ protocol to ensure the scientific rigor and accuracy of the testing.

After the tests, participants entered a 12-month follow-up period during which non-contact low back injuries were monitored. Injury incidents were recorded by the rescue team’s squadron leader and submitted to the professional sports trainer responsible for team care for assessment.

According to the International Olympic Committee (IOC) Consensus Statement (23), the following characteristics were documented for each injured rescue team member: the affected body part and the duration of missed training or rescue operations. Non-contact injuries were defined as any non-contact muscular, joint, or skeletal issues, or injuries to the lumbar region and lower limbs (classified by area: lumbar region, buttocks, thigh, knee, leg, ankle, or foot) that occurred during routine training or rescue operations. Additionally, non-contact injuries had to fulfill the following criteria: the injury occurred while participating in organized routine physical training; it required medical attention; and it necessitated the rescue team member’s withdrawal from that day’s training or absence from subsequent routine training and rescue operations (24). Injuries occurring during non-routine training or rescue operations are excluded. Researchers reviewed and collated injury records weekly to ensure data accuracy. If a rescue team member sustained multiple injuries during the follow-up period, only the first injury was included in the study.

### 2.3 Statistical analysis

To quantify asymmetry in the SLCMJ test, the study employed the formula proposed by Bishop et al. (25) for unilateral tasks: (A = the maximum value of the functional index in the left or right leg, B = the minimum value of the functional index in the left or right leg).


Index=∣A−B∣/∣A∣×100%

This formula calculates the absolute percentage difference between limbs, irrespective of which limb is dominant or non-dominant. Therefore, the analysis did not pre-specify or differentiate based on limb dominance (e.g., kicking leg); it treated asymmetry as a direction-agnostic, magnitude-based risk factor. The use of absolute values ensures the index is always positive.

This study adopted a predictive modeling approach rather than a causal inference framework. The primary goal was to identify factors associated with injury risk that could be useful for screening purposes.

First, the normality of continuous data (demographics, asymmetry indices) was assessed using the Kolmogorov–Smirnov test (*p* < 0.05), and homogeneity of variance was checked with Levene’s test (*p* < 0.05). Variables following a normal distribution were compared using independent t-tests; otherwise, the Mann–Whitney U test was used (e.g., for between-group comparisons of demographics and asymmetry indices, *p* < 0.05).

Second, univariate log-binomial regression analyses were conducted to assess the crude association between each asymmetry index and non-contact low back injury risk. Where the log-binomial model failed to converge, Poisson regression with robust standard errors was used as a reliable alternative to estimate risk ratios (*p* < 0.05). Indicators with *p* < 0.1 in univariate analysis were considered candidate variables for multivariable analysis.

Third, to build the final predictive model, multicollinearity among candidate variables and potential confounders (age, height, weight, BMI, length of service) was assessed using the variance inflation factor (VIF). Variables with VIF > 5 were considered indicative of substantial multicollinearity and were excluded. To assess the potential impact of unmeasured confounding on the observed associations between asymmetry indices and injury risk, E-values were calculated for significant risk estimates. The retained variables were entered into a multivariable Poisson regression model with robust standard errors to identify independent risk factors, presented as RR with 95% confidence intervals (CI). Absolute risk differences (RD) and their 95% CIs were calculated separately using a linear probability model with robust standard errors, complementing the RR estimates. The Hosmer-Lemeshow (*p* > 0.05) and Omnibus (*p* < 0.05) tests were used to evaluate the multivariate regression model.

For the asymmetry index that demonstrated a statistically significant association with non-contact low back injuries in the multivariate Log-binomial or Poisson regression analysis, a receiver operating characteristic (ROC) curve was employed to establish the optimal cut-off point for injury prediction. The optimal cut-off point on the ROC curve was determined as the Youden index (Youden index = sensitivity + specificity − 1) yielding the maximum combined sensitivity and specificity. Participants were then categorized into high- and low-risk groups based on ROC curve cut-off point, and the RR and RD for injury between these groups were calculated using Poisson and linear probability models, respectively. All statistical analyses were performed using SPSS 26.0 (IBM Corp., Armonk, NY, USA), with a pre-specified alpha level of 0.05. Participants with missing injury reports or data were excluded from the analyses.

## 3 Results

During the 12-month follow-up period, 15 out of the 87 team members (17.2%) sustained non-contact low back injuries. Among these 15 injuries, the majority were primarily Chronic Lumbar Muscle Strain (*n* = 11), with Acute Lumbar Muscle Strain (*n* = 4). The median time lost from training or duty due to injury was 7 days (interquartile range: 5–14 days). The basic characteristics of the team members are presented in Table 1. No significant differences were observed between the injured and uninjured members in terms of age, length of service, height, or weight (*p* > 0.05).

**Table 1:** Characteristics of mine rescue workers.^a^

Basic information	With lumbar injury (Mean ± SD)	*n*	No lumbar injury (Mean ± SD)	*n*	*p*
Height (cm)	173.8 ± 5.1	15	174.2 ± 5.0	72	0.714
Weight (kg)	75.8 ± 9.3	15	76.0 ± 10.4	72	0.980
Age (years)	36.9 ± 4.7	15	35.9 ± 4.6	72	0.444
Length of service (years)	14.0 ± 6.2	15	12.7 ± 4.8	72	0.759
BMI (kg/m^2^)	24.6 ± 3.5	15	24.5 ± 2.9	72	0.957

a *p*-values indicate statistical significance (Mann–Whitney U, *p* < 0.05).

As an initial exploratory step to identify candidate predictors from the multiple asymmetry metrics collected, univariate regression analyses were performed. The log-binomial model failed to converge for several variables; therefore, Poisson regression with robust standard errors was employed consistently for all univariate analyses, as per the pre-specified analytical plan. This analysis identified four asymmetry indicators with *p* < 0.1 for inclusion in subsequent multivariable modeling: eccentric peak force (N), vertical velocity at takeoff (m/s), peak takeoff acceleration (N), and takeoff peak force (N). Details are presented in Table 2.

**Table 2:** Poisson regression analysis of bilateral asymmetry of lower limbs of mine rescue workers.^a^

Target	*n*	Mean ± SD (%)	RR (95%CI)	*p*
Eccentric peak force	87	9.16 ± 7.12	0.912 (0.828, 1.005)	0.063
Peak takeoff acceleration	87	6.61 ± 5.49	0.954 (0.905, 1.006)	0.084
Takeoff peak force	87	5.84 ± 3.68	1.302 (1.092, 1.552)	<0.001
Vertical velocity at takeoff	87	7.81 ± 6.53	0.947 (0.924, 0.983)	0.006

a *p*-values indicate statistical significance (*p* < 0.1), and the values of each index are bilateral asymmetry indices (%). RR = Risk Ratio; CI = Confidence Interval.

To identify the most discriminative indicator for subsequent multivariate analysis, the bilateral asymmetry indices of these four indicators were compared between the injured and uninjured groups using the Mann–Whitney U test (Table 3).

**Table 3:** Bilateral asymmetry of indicators related to the lower limbs of mine rescue workers^a^ (median, quartiles).

Target	With lumbar injury	*n*	No lumbar injury	*n*	*p*
Eccentric peak force	3.45 (1.575, 9.85)	15	8.55 (4.075, 14.85)	72	0.111
Peak takeoff acceleration	7.9 (4.325, 12.45)	15	11.1 (5.425, 18.8)	72	0.228
Takeoff peak force	7.7 (5.4, 9.25)	15	4.95 (2.525, 8.35)	72	0.007
Vertical velocity at takeoff	7.45 (4.1, 12.125)	15	6.35 (3.175, 9.4)	72	0.249

a Outside center parentheses is the median, inside center parentheses is the rank in 25 and 75% quartiles in that order, *p* values indicate statistically significant differences between groups with and without low back injuries (Mann–Whitney U, *p* < 0.05).

The results revealed that, among all indicators, only the asymmetry of takeoff peak force showed a statistically significant difference between the two groups. The injured group exhibited a significantly higher degree of asymmetry in takeoff peak force compared to the uninjured group (mean rank: 41.01 vs. 60.22; *Z* = −2.721, *p* = 0.007). Based on this result, the bilateral asymmetry of takeoff peak force was selected as the primary variable for further investigation into the risk of non-contact low back injuries.

The study assessed potential variables for multicollinearity. First, any factor with a variance inflation factor (VIF) greater than 5 was excluded. Second, based on a comparison of the −2 log-likelihood values between nested models, the following variables were also excluded: height, weight, BMI, peak acceleration at jump, peak centrifugal force, and concentric impulse. Consequently, the following variables were included in the final multifactor Poisson regression model: vertical velocity at takeoff, takeoff peak force, weight, and length of service.

In the final multivariate Poisson regression model (Table 4), multicollinearity was assessed. The VIF values for each variable were as follows: takeoff peak force (VIF = 1.106), vertical velocity at takeoff (VIF = 1.088), weight (VIF = 1.014), and length of service (VIF = 1.028). All VIF values were well below the exclusion threshold of 5, indicating no serious multicollinearity among the factors and that redundant information would not be introduced for predicting the dependent variable. The Hosmer-Lemeshow test was not significant (*χ*^2^ = 13.052, df = 8, *p* = 0.110), indicating a good model fit. The Omnibus test of model coefficients was significant (*χ*^2^ = 12.324, df = 4, *p* = 0.015), indicating a significant difference between the model and the null hypothesis model.

**Table 4:** Poisson regression analysis of risk factors for non-contact low back injuries.^a^

Risk factors	*RR* (95%CI)	*RD* (95%CI)	*p*
Takeoff peak force	1.185 (1.091, 1.288)	0.032 (0.009, 0.056)	<0.001
Vertical velocity at takeoff	1.027 (0.983, 1.073)	0.008 (−0.004, 0.021)	0.227
Weight	0.987 (0.939, 1.037)	−0.002 (−0.01, 0.006)	0.595
Length of service	1.031 (0.975, 1.095)	0.008 (−0.007, 0.024)	0.288

a *p*-values indicate the association between each risk factor and low back injury in a multifactorial Poisson regression model (*p* < 0.05), and the values of each metric are two-sided asymmetry indices (%).

Based on this, the ROC curve for the asymmetry of takeoff peak force was analyzed (AUC = 0.731; 95% CI: 0.614–0.847, *p* = 0.004), indicating a moderate predictive ability. The optimal threshold, determined by the Youden’s index, was 7.05% (sensitivity = 0.688, specificity = 0.718). Using this cut-off point, participants were categorized into two groups: those with a takeoff peak force asymmetry greater than 7.05% were defined as the high-risk group, and those with asymmetry of 7.05% or less were defined as the low-risk group. This continuous variable was thus transformed into a dichotomous variable for subsequent analysis. The data were input into the final multivariate Poisson regression model. The results showed that the high-risk group was significantly associated with non-contact lumbar injury (Table 5).

**Table 5:** Poisson regression analysis of risk-stratified for non-contact low back injuries.^a^

Risk factors	*RR* (95%CI)	*RD*	*p*
Takeoff peak force	3.647 (1.353, 9.832)	0.241 (0.073, 0.409)	0.011
Vertical velocity at takeoff	1.033 (0.997, 1.071)	0.010 (−0.003, 0.005)	0.075
Weight	0.982 (0.935, 1.032)	−0.002 (−0.010, 0.005)	0.475
Length of service	1.035 (0.975, 1.098)	0.009 (−0.007, 0.024)	0.256

a *p*-values indicate the association between each risk factor and low back injury in a multifactorial Poisson regression model (*p* < 0.05), and the values of each metric are two-sided asymmetry indices (%).

## 4 Discussion

This prospective cohort study aimed to investigate the relationship between bilateral asymmetry in lower limb explosive strength and the risk of non-contact low back injuries among mine rescue personnel. Additionally, it sought to identify predictive factors for these injuries. The findings are crucial for developing scientifically sound prevention strategies and training programs. The results indicate that peak vertical jump force metrics in the SLCMJ test are significantly correlated with non-contact low back injuries. As shown in Table 3, the degree of asymmetry in peak vertical jump force among rescue personnel who sustained low back injuries was significantly higher than among those who did not (*p* = 0.007). According to the results of the multifactorial Poisson regression analysis (Table 4), bilateral asymmetry in peak jump force was the sole risk factor. Specifically, for each 1% increase in takeoff peak force asymmetry, the relative risk of lumbar injury increased by approximately 18.5% (RR = 1.185, 95% CI: 1.091–1.288), which corresponds to an absolute risk increase of 4.7%. More practically, when using the identified 7.05% screening threshold, rescue workers classified as high-risk faced a near four-fold increase in relative risk (RR = 3.647) and an absolute injury incidence that was 24.1% higher than their low-risk counterparts. These risk-based metrics (RR and RD) provide clearer and more actionable information for prevention program design, as OR tends to exaggerate the apparent strength of the association between exposure and outcome (whether positive or negative) compared to RR (26). In summary, the takeoff peak force during the SLCMJ test is a predictive risk factor for non-contact low back injuries among mine rescue personnel.

To assess the potential impact of unmeasured confounding on the observed associations, we calculated E-values for the primary risk estimates. For the association per 1% increase in continuous asymmetry (RR = 1.185, 95% CI: 1.091–1.288), the E-value for the point estimate was 1.58, and for the lower confidence limit (RR = 1.091) it was 1.29. For the association based on the dichotomized asymmetry (high-risk vs. low-risk group, RR = 3.647, 95% CI: 1.353–9.832), the E-value for the point estimate was 7.27, and for the lower confidence limit (RR = 1.353) it was 1.94.

In addition to relative risk (RR), we reported risk differences (RD) to convey absolute risk increases. For each 1% increase in asymmetry, the absolute risk of injury rose by 3.2% (RD = 0.032). When using the 7.05% cutoff, high-risk individuals had an absolute injury rate 24.1% higher than low-risk individuals. These RD estimates are clinically informative, as they quantify the actual burden of injury attributable to asymmetry and can guide the prioritization of interventions.

This study aligns with the broader literature linking inter-limb asymmetry to injury risk, yet the optimal asymmetry threshold of 7.05% is notably lower than the 10–15% often cited in sports medicine research (27–30). This discrepancy underscores the critical influence of population-specific characteristics. Brumitt et al. (27) suggested that optimal thresholds may vary across athletic populations. This perspective directly extends to specialized occupational groups like mine rescuers For instance, while a Y-balance test (YBT) reach asymmetry threshold of 4 cm is often reported for injury prediction in athletic populations (8, 31, 32), research by Vaulerin et al. (15) identified a lower optimal threshold (2–3 cm) for firefighters. Therefore, the optimal thresholds established in general research may not be directly applicable to mine rescue personnel due to their unique characteristics (e.g., age, weight), demanding working environment (e.g., high pressure, high temperature), and the complex, hazardous nature of their duties. However, as this study was conducted within a single rescue squadron in China, the generalizability of the 7.05% threshold requires further validation.

Bilateral asymmetry in lower limb strength and power is commonly assessed using isometric/isokinetic muscle strength tests or unilateral/bilateral jump tests (17). For example, Hietamo et al. (33) evaluated lower limb asymmetry by measuring peak torque during isometric testing. Other studies have used metrics such as single-leg countermovement jump height or distance to calculate asymmetry and validate its association with injury (34–36). Similarly, Read et al. (7) identified peak landing vertical ground reaction force as a strong predictor of injury in football players. Correspondingly, the present analysis revealed that takeoff peak force also has good predictive efficacy for non-contact low back injuries in mine rescue personnel. It is widely used to assess lower limb muscular capabilities, such as explosive power and absolute strength (37).

Previous research has suggested that factors like training history and body mass index (BMI) can influence injury risk. For instance, Guan et al. (38) reported an increased risk of lower limb sports injuries with accumulated training years in adolescent athletes, and Kobayashi et al. (39) highlighted BMI’s significant impact on lateral ankle sprain risk. In the present study, however, the multivariate Poisson regression model—after screening for and adjusting multicollinearity—revealed that neither body weight (*p* = 0.584) nor length of service (*p* = 0.358) was significantly associated with non-contact low back injuries. In contrast, the bilateral lower limb takeoff peak force asymmetry index was significantly correlated with these injuries (*p* = 0.013).

According to Cheng et al. (2), sports injuries constitute one cause of functional asymmetry in athletes’ lower limbs. Consequently, a history of previous injuries may also represent a potential risk factor. However, given that the subjects’ average length of service was 12.97 years—an excessively long period to accurately gather details of their past injuries—a history of previous injuries was not included in this study. Future research should investigate the duration of injuries among specialized occupational groups, incorporating prior injury histories into analyses to yield more accurate findings. Brumitt et al. (11, 27) incorporated training and match duration (i.e., sports exposure time) as a risk factor in their risk prediction study for female university volleyball players. The inclusion of exposure time effectively controls and filters for confounding variables, enhancing model accuracy and enabling more precise quantification of risk probabilities. Simultaneously, it standardizes injury occurrence by expressing it as injuries per 1,000 h of exposure time, facilitating objective analysis of injury trends over time.

This study focused on bilateral asymmetry in lower limb explosive strength and did not assess other potential factors like flexibility or balance. Future research could incorporate assessments such as the YBT, Star Excursion Balance Test (SEBT), and range of motion (ROM) measurements to provide a more comprehensive analysis of lumbar injury risk factors. Combining these with lower limb explosive strength testing could lead to the development of robust risk prediction indicators. The practical application of asymmetry measures is supported by studies such as Paterno et al. (40), who used bilateral lower limb strength asymmetry as a criterion for return-to-play decisions in ACL reconstruction. Similarly, bilateral lower limb asymmetry could serve as one of the key criteria for determining when mine rescue personnel are fit to return to training and duty following lumbar or other non-contact injuries, thereby safeguarding their long-term health.

## 5 Limitations

This study did not statistically analyze the training and working hours of mine rescue personnel as potential risk factors for inclusion in the analysis. However, as all personnel were drawn from the same rescue squadron, their rescue training and duties exhibited homogeneity, thereby controlling for the relevant impact of this factor to a certain extent. Nevertheless, future research should incorporate statistical data on subjects’ training and working hours, alongside documentation of the stage and timing of injury occurrence, to enhance the study’s accuracy.

As all participants in this study were drawn from the same region, their risk factor profiles may be influenced by shared living environments and occupational duties. In addition, as this study was a single-center study conducted within a specific mine rescue team in China, and there may be sampling bias, it is difficult to accurately reflect the actual situation of rescue teams in other regions. Future research should incorporate heterogeneity analyses, including rescue personnel from diverse regions and units, to better reflect the injury risk factors across the entire mining rescue community.

The estimates derived from regression models (e.g., RR, RD) are presented as associational measures and should not be interpreted as causal effects. While the study adjusts for several covariates, unmeasured confounding may still exist. The selection of covariates was guided by both statistical criteria (e.g., VIF) and subject-matter relevance (e.g., weight, length of service) to optimize model stability and predictive performance, rather than to fully establish causality.

To assess the potential impact of selection bias due to loss to follow-up, baseline characteristics (age, height, weight, length of service, and baseline takeoff peak force asymmetry) were compared between participants who completed the study and those lost to follow-up using Mann–Whitney U tests. No significant differences were found (all *p* > 0.10), suggesting that the data may be missing at random (MAR) with respect to these measured variables. Given the relatively low proportion of loss to follow-up (13.9%) and the lack of evidence for systematic differences in key baseline measures, primary analyses were conducted on the complete-case cohort (*n* = 87). The assumption of non-informative censoring, while not verifiable for unmeasured factors, is considered plausible in this context. For sensitivity, inverse probability of censoring weights (IPCW) were considered but not applied in the primary model, as the conditions for MAR appeared reasonably met and the added complexity was deemed unnecessary for the primary predictive aim of this study. However, the potential for residual selection bias cannot be entirely ruled out.

## 6 Conclusion

Assessment of bilateral asymmetry in lower limb explosive force indexes (e.g., peak force of jump) effectively evaluates the risk of non-contact low back injuries in mine rescue personnel. These findings supplement existing risk factors for mine rescue workers, providing evidence for applying lower limb asymmetry assessments in specialized occupations such as emergency rescue. This offers healthcare professionals and physical trainers in relevant fields additional options for developing comprehensive, systematic preventive measures and training programs.

Future research should incorporate additional limb asymmetry assessments alongside potential risk factors such as prior injuries and exposure duration to further explore the relationship between bilateral asymmetry in lower limb explosive strength and non-contact low back injuries.

## Data Availability

The original contributions presented in the study are included in the article/supplementary material, further inquiries can be directed to the corresponding author.
